# xMEN: a modular toolkit for cross-lingual medical entity normalization

**DOI:** 10.1093/jamiaopen/ooae147

**Published:** 2024-12-26

**Authors:** Florian Borchert, Ignacio Llorca, Roland Roller, Bert Arnrich, Matthieu-P Schapranow

**Affiliations:** Hasso Plattner Institute for Digital Engineering, University of Potsdam, Potsdam 14482, Germany; Hasso Plattner Institute for Digital Engineering, University of Potsdam, Potsdam 14482, Germany; Speech and Language Technology Lab, German Research Center for Artificial Intelligence (DFKI), Berlin 10559, Germany; Hasso Plattner Institute for Digital Engineering, University of Potsdam, Potsdam 14482, Germany; Hasso Plattner Institute for Digital Engineering, University of Potsdam, Potsdam 14482, Germany

**Keywords:** clinical natural language processing, entity linking, multilingual, Unified Medical Language System, Snomed CT

## Abstract

**Objective:**

To improve performance of medical entity normalization across many languages, especially when fewer language resources are available compared to English.

**Materials and Methods:**

We propose xMEN, a modular system for cross-lingual (x) medical entity normalization (MEN), accommodating both low- and high-resource scenarios. To account for the scarcity of aliases for many target languages and terminologies, we leverage multilingual aliases via cross-lingual candidate generation. For candidate ranking, we incorporate a trainable cross-encoder (CE) model if annotations for the target task are available. To balance the output of general-purpose candidate generators with subsequent trainable re-rankers, we introduce a novel rank regularization term in the loss function for training CEs. For re-ranking without gold-standard annotations, we introduce multiple new weakly labeled datasets using machine translation and projection of annotations from a high-resource language.

**Results:**

xMEN improves the state-of-the-art performance across various benchmark datasets for several European languages. Weakly supervised CEs are effective when no training data is available for the target task.

**Discussion:**

We perform an analysis of normalization errors, revealing that complex entities are still challenging to normalize. New modules and benchmark datasets can be easily integrated in the future.

**Conclusion:**

xMEN exhibits strong performance for medical entity normalization in many languages, even when no labeled data and few terminology aliases for the target language are available. To enable reproducible benchmarks in the future, we make the system available as an open-source Python toolkit. The pre-trained models and source code are available online: https://github.com/hpi-dhc/xmen

## Introduction

**Figure 1.**
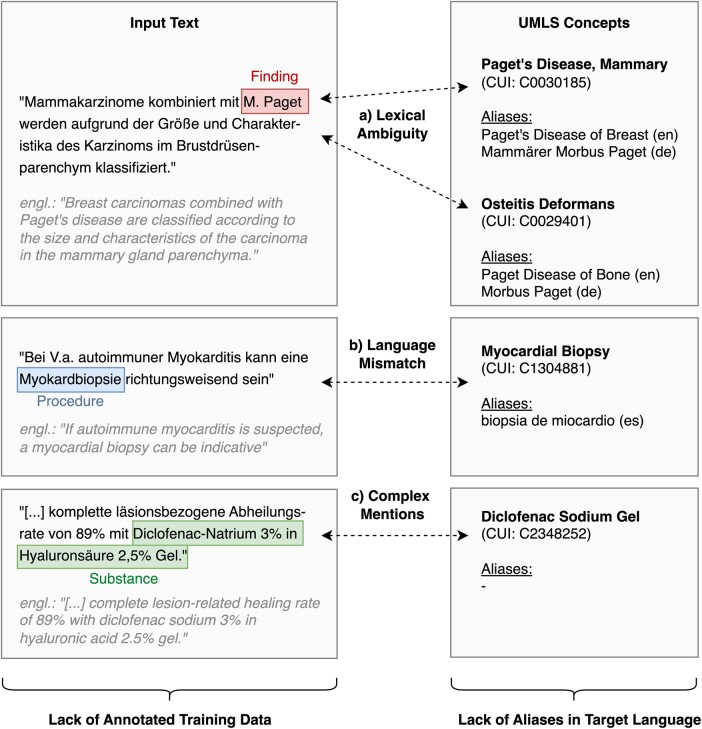
Challenges for cross-lingual medical entity normalization, input text examples in German (with English translations).

Extraction of named entities from free-text documents is a core component in most medical natural language processing (NLP) pipelines. An important sub-task is the normalization of identified entity mentions to canonical identifiers in a controlled vocabulary, ontology, or other terminology system, such as the Unified Medical Language System (UMLS), to ensure semantic interoperability.^1^ Medical Entity Normalization (MEN) is challenging for multiple reasons, illustrated in Figure 1.

A frequently encountered problem in general-domain and medical entity normalization is *lexical ambiguity*: as a well-known example, the expression “Paget’s disease” may refer to either *Paget’s disease of the bone* (“Osteitis Deformans”, UMLS CUI: C0029401), *Paget’s disease of the breast* (“Paget’s Disease, Mammary”, C0030185), or *extramammary Paget’s disease* (“Paget Disease Extramammary”, C0030186).^2^ Therefore, the correct disambiguation of a mention of “Paget’s disease” depends on its context. In medicine, an additional problem is the potentially large number of surface forms and synonyms for a given concept^3^; this usually renders an enumeration of all potential aliases infeasible. This is an even larger challenge for MEN in non-English contexts: most concept aliases in terminology systems like the UMLS are only available in English,^4^ limiting the applicability of approaches that rely on such aliases to match entity mentions to concepts. For instance, when trying to normalize the mention “Myokardbiopsie” in German text, no aliases in the target language that could be used for surface form matching of this rather simple mention, are currently available in the UMLS. The problem is exacerbated in the presence of *complex entity mentions*, consisting of multiple tokens, where lexical overlap with curated aliases diminishes quickly. Moreover, recent state-of-the-art MEN approaches are based on supervised machine learning, thus requiring training corpora annotated with grounded entity mentions. Again, such annotation projects have been carried out predominantly for English corpora.

In this work, we propose a modular, Transformer-based approach for cross-lingual (x) MEN to address these challenges and effectively leverage all resources available for a particular language, dataset, and task. Following the general *generate-and-rank* architecture described by Sevgili et al,^5^ we first identify potentially matching concepts in a target knowledge base (KB) for a given mention span in a candidate generation (CG) step. Second, we adapt the ranking to the specific task, also incorporating contextual information. When sufficient aliases in the target language are unavailable, our cross-lingual CG approach uses aliases in other languages (usually English). For subsequent task-specific re-ranking, we train cross-encoder (CE) models in various languages. To effectively combine state-of-the-art CG approaches with subsequent re-ranking, we propose a new loss function for training CE models. For domains without sufficient training data, we create weakly supervised (WS) models based on large-scale datasets obtained through neural machine translation (NMT) and label projection. In the high-resource scenario, that is, when sufficient annotated examples are available, we can train fully supervised (FS) CE models instead of the pre-trained ones.

We evaluate our cross-lingual medical entity normalization (xMEN) pipeline across a diverse set of benchmark datasets in English, Spanish, French, German, and Dutch. Leveraging the different kinds of information available for each dataset and language, we obtain new state-of-the-art results in most cases. We focus our evaluation on this selection of European languages based on recent literature reviews, as we require at least a small amount of high-quality gold-standard test data for a meaningful performance benchmark.^6–8^

The remainder of this work is organized as follows: we begin by setting our contributions in the context of related work, followed by a description of the methods implemented in xMEN. We then share the experimental setup and results for our benchmarks. Finally, we discuss our findings and conclude our work with an outlook.

## Background and significance

Many widely used standalone tools that implement MEN, such as cTAKES,^9^  MetaMap,^10^  QuickUMLS,^11^ or scispaCy^12^ are based on elaborate forms of dictionary matching. Although they provide robust performance, they implicitly assume the availability of concept aliases in the target language and are usually applied to English text only. More recent research prototypes, such as the systems proposed by Sung et al,^13^ Bhowmik et al,^14^ Agarwal et al,^15^ or Yuan et al^16^ rely on datasets with sufficient annotations of grounded entity spans. These datasets are predominantly available in English—some widely used examples are MedMentions,^17^ NCBI-Disease,^18^  BioCreative V CDR,^19^ or COMETA.^20^ For a recent survey of such systems and corpora, please refer to French and McInnes^7^ or Garda et al.^21^

Several approaches have been proposed to address the scarcity of annotated data and concept aliases for non-English languages. Recent works leverage cross-lingual synonym relationships and other assertions from the UMLS for representation learning: Wajsbürt et al^22^ present MLNorm, a system that leverages French and English synonyms for training a distantly supervised Bidirectional Encoder Representations from Transformers (BERT) classification model. The authors achieve competitive performance on the Quaero corpus,^23^ which can be further improved with a FS model, using gold-standard annotations. To learn concept representations irrespective of the target task, Liu et al^24^ propose SapBERT, a BERT model that has been further fine-tuned on UMLS synonyms to improve the representation of semantic similarity in the BERT embedding space. Using additional UMLS relations besides synonymy, Yuan et al^25^ propose CODER for learning multilingual concept representations. Their approach is evaluated across different tasks that measure semantic similarity, including MEN on the multilingual Mantra Gold Standard Corpus (GSC).^26^ Our proposed pipeline uses such representations for unsupervised, cross-lingual CG, but combines it with a FS or WS re-ranker. In particular, we use the cross-lingual version of SapBERT for CG, which has shown competitive performance on several biomedical EL benchmarks, even without fine-tuning on task-specific data.^27^^,^^28^

A different approach for leveraging scarce resources is based on machine translation. Roller et al^29^ propose a multistep system for dictionary-based candidate retrieval, combining a direct lookup using both UMLS aliases in the target language and English with a cross-lingual lookup of the machine-translated mention in the UMLS. The authors achieve competitive performance on the Quaero and Mantra GSC corpora. Our method also uses NMT for leveraging English-language resources. However, instead of translating mentions for the target task, we apply NMT to obtain large-scale, weakly labeled datasets in the target language and use this data to initialize a supervised, language-specific re-ranker, which can be easily shared and used across tasks. The strategy to leverage entity annotations from a high-resource language through NMT has been successfully applied for other medical information extraction tasks, such as named entity recognition (NER).^30–32^

In addition to improvements in performance, our work addresses several concerns that have been noted to impact the validity and reproducibility of existing benchmarks.^7^^,^^33^ These include the implementation of evaluation metrics, data pre-processing steps, and the reproducible definition of target KBs. To this end, we make our system available as an extensible Python library. The xMEN toolkit can be used with any dataset that follows the BigBIO schema, which can be readily obtained from most span-based annotation formats and has been implemented for a wide range of MEN benchmark datasets.^34^

The contributions of our work can be summarized as follows:

- Improvement of entity normalization performance in low-resource scenarios through cross-lingual CG and WS pre-raining of re-rankers, building upon recent advances in NMT and label projection,
- A novel technique called *rank regularization* to combine the ranking suggested by a general-purpose CG component with task-specific re-rankers,
- New state-of-the-art performance on various non-English MEN benchmark datasets,
- An easy-to-use and modular open-source Python toolkit for language-independent MEN that integrates seamlessly with existing language resources, such as the BigBIO framework and common NER tools, as well as
- A framework for reproducible benchmarks with explicit configuration of task-specific sets of target concepts and transparent evaluation criteria.

## Methods

**Figure 2.**
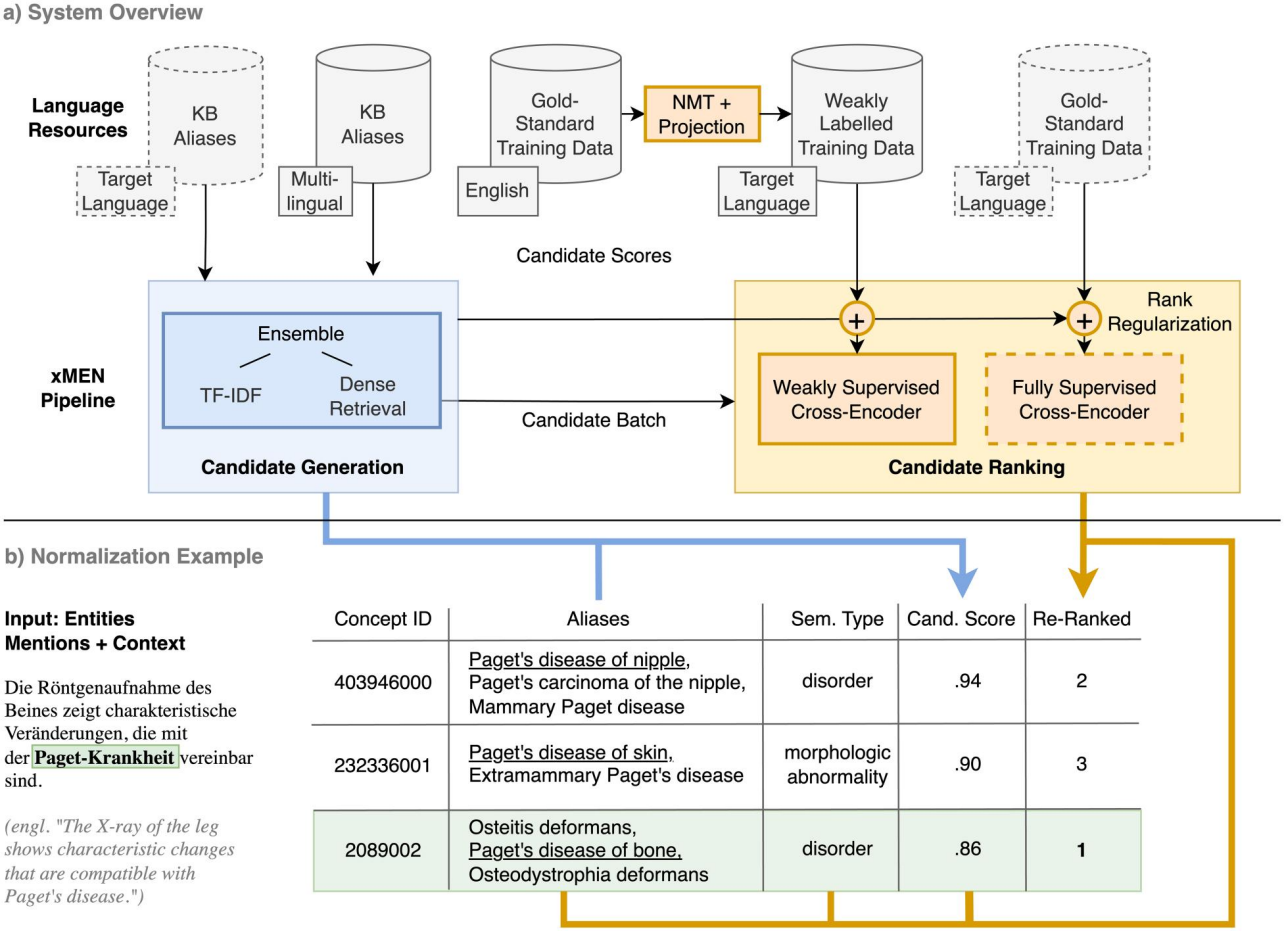
Overview of (A) our entity normalization system along with (B) a normalization example (based on Snomed CT concepts). Most existing MEN approaches assume KB aliases in the target language, as well as gold-standard training data for supervised model training (dashed borders). While our system benefits from these, it does not rely on them: when sufficient aliases in the target language are unavailable, cross-lingual CG incorporates aliases in other languages. For candidate ranking, we propose to use WS models when FS ones cannot be trained due to the lack of training data.

In the following, we describe the main components of the xMEN system, presenting the components in the order they are used in later experiments. An overview of the system is shown in Figure 2. Our main methodological innovations concern the candidate ranking step, while relying on well-established unsupervised methods for CG. The application of these methods through the xMEN Python toolkit is described at the end of this section.

### Terminology systems and knowledge bases

In the biomedical domain, the goal of entity normalization is usually to link mentions to a concept identifier from a controlled vocabulary, ontology, or other medical terminology system. As MEN can be performed against any such system, we use the term *knowledge base* (KB) in a task-agnostic manner. For our purposes, a KB includes a list of biomedical concepts of interest together with their metadata, like aliases, semantic type information, or definitions. In our experiments, target KBs are task-specific subsets of the UMLS,^1^  Snomed CT,^35^ as well as German versions of ICD-10,^36^ OPS,^37^ and ATC,^38^ depending on the benchmark dataset. Additional aliases for KB concepts are obtained from different sources, particularly when few aliases in the target language are available. For instance, we obtain (mostly English) aliases from DrugBank^39^ for the German version of ATC in our experiments, as described in File S1.

### Pre- and post-processing

For some corpora, each span is annotated with a semantic class in addition to the gold-standard concept identifier (e.g., a UMLS semantic group), and a mapping between these classes and types in the target KB is often possible. Similarly, most NER taggers classify detected entity spans into such classes. In these cases, the lists of predictions can be restricted to such compatible concepts.^22^^,^^29^ In addition, we incorporate a simple abbreviation expansion, using the algorithm proposed by Schwartz and Hearst^40^ and an implementation from scispaCy.^12^

### Candidate generation and ranking

Potential candidates for entity mentions in target KBs can be identified using different approaches. In our experiments, we use an ensemble of a TF-IDF vectors over character n-grams for surface form matching, and embeddings obtained from the cross-lingual version of SapBERT.^27^ These encoding methods are applied to all aliases in the target KB to create an index. For inference, the same encoding is applied to mention spans, followed by an approximate nearest neighbor search to generate a ranked list of *k* candidates. For all experiments, we use *k *=* *64 candidates, consistent with prior work.^41^

The ranking induced by this unsupervised CG step is independent of the dataset and its annotations. To adapt the ranking for specific tasks, we use a CE with representations of mentions with their contexts and target concepts. In contrast to the CG, the CE is trainable and uses the ground-truth concept labels in the training data. Details regarding the different CG implementations and the mention/context encodings for re-ranking are provided in File S2.

### Training cross-encoders with rank regularization

For training the CE, training batches are constructed for each mention and *k* candidate concepts. As the input representation for each pair of a mention *m* and candidate concept *e*, we concatenate the mention-context and concept encoding. We then feed these inputs through a BERT-based encoder with a linear output layer, assigning a score *s*(*m*, *e*) to each such mention-concept pair. In each batch, we include a synthetic NIL (not-in-list) concept encoded as [UNK] to enable the model to abstain from a prediction in cases, where the correct concept is not among the candidates.

We train the CE model using Sentence Transformers framework^42^ to maximize the score of the correct candidate $e_{i}$ within each batch given a softmax loss $L_{sm}(m,e_{i})$. However, using this loss alone neglects the potentially meaningful ranking suggested by the prior CG step. Therefore, we introduce a new *rank regularization* term to the loss function, pushing the logits of the classification layer to preserve the original ranking while still maximizing top-1 accuracy. Formally, our new loss function is defined as:

$$L(m,e,y)=\sum_{i=1}^{k}y_{i}L_{sm}(m,e_{i})+\lambda ||s(m,e)-c(m,e)|{|}_{2}$$

with **y** defining the vector of one-hot-encoded ground truth labels per batch, c(m,e) is the vector of scores assigned by the CG, and λ is a hyperparameter controlling the trade-off between top-1-accuracy and preservation of the original ranking. The standard softmax loss for a single candidate (as described by Wu et al^41^) is given by:

$$L_{sm}(m,e_{i})=-s(m,e_{i})+\log\sum_{j=1}^{k}exp(s(m_{i},e_{j}))$$

We set λ=1.0 in all experiments, based on a hyperparameter search to optimize validation set performance (see File S1). A detailed analysis of the impact of rank regularization is presented in the Results section.

### Weakly labeled datasets for cross-encoder training

To obtain CE models for target languages where no training data with grounded entity mentions is available, we propose a *weakly supervised* approach. To this end, we transfer the entity annotations from a high-resource language (usually English) using NMT and projection of the original mention spans. We consider this to be a weak supervision signal, as the resulting labels are noisy. Our implementation builds upon the recently proposed EasyProject method, an open-source model for simultaneously performing NMT and entity alignment.^43^ It shows remarkably good performance by simply inserting special markers (like brackets []) around entity mentions in the source text and translating the tagged text. In our experiments, we use the checkpoint ychenNLP/nllb-200-3.3b-easyproject from the Hugging Face Hub, which was fine-tuned on synthetic data to preserve markers more reliably than the original model.^44^

In our experiments, we translate MedMentions (ST21pv),^17^ the largest English-language MEN dataset with more than 200K grounded entity mentions, to French, Spanish, German, and Dutch. The translated datasets are then used to train a supervised CE model with rank regularization, as described in the previous section. These pre-trained models are made available on the Hugging Face Hub. This process can be easily repeated for any language supported by EasyProject.

### The xMEN Python toolkit

**Figure 3.**
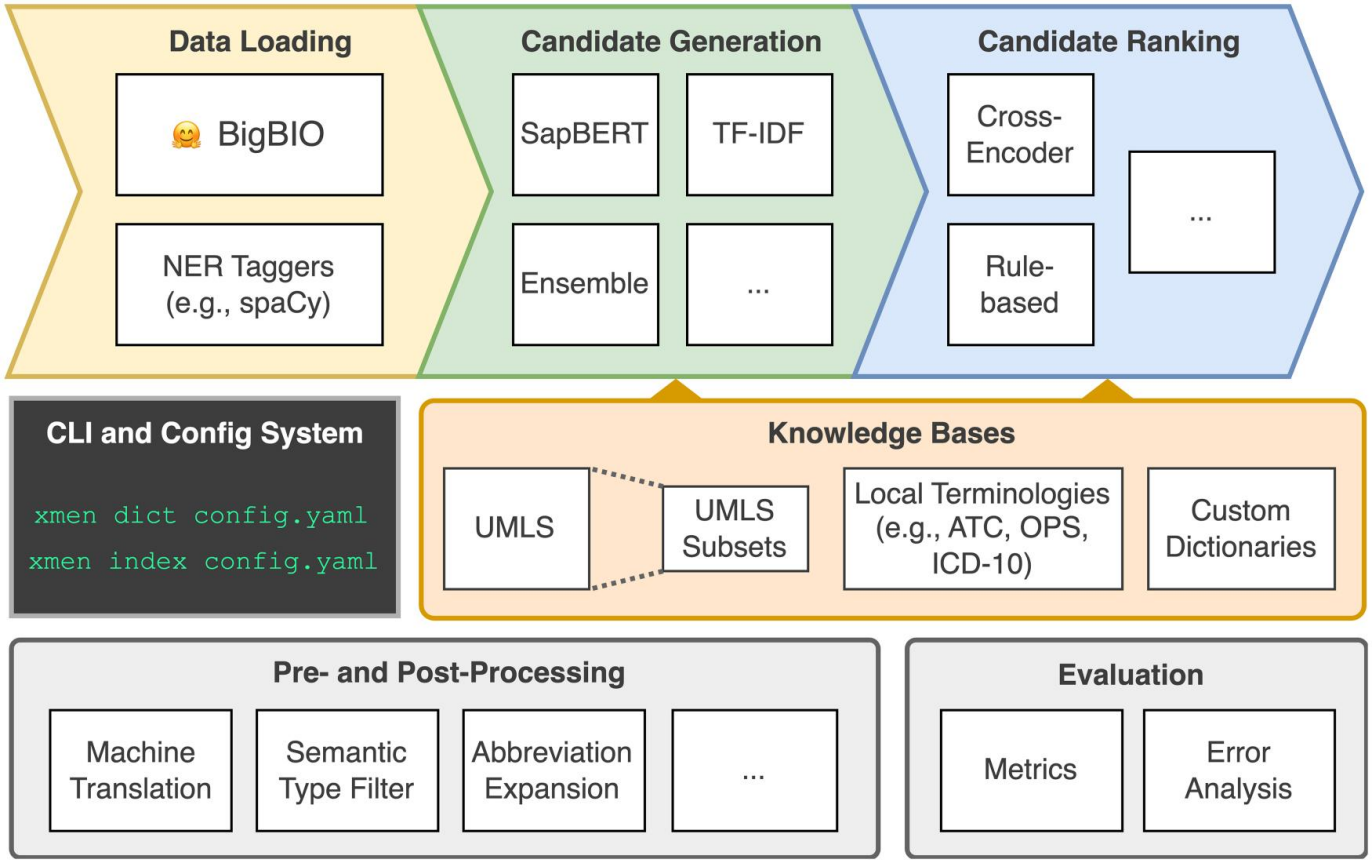
Overview of the modular architecture of the xMEN open-source Python toolkit. It can be used with any BigBIO-compatible dataset and implements different approaches for CG and ranking, pre- and post-processing steps, evaluation metrics and utilities for error analysis. In addition, different KBs can be quickly integrated and indexed via the configuration system and CLI. New modules can be easily integrated (denoted by boxes with 3 dots).

All aforementioned methods and functionalities are accessible through the open-source xMEN toolkit, an overview of which is given in Figure 3. It provides a Python API and a command-line interface (CLI) with Yaml-based configuration files, that is, a human-readable markup language. As most MEN benchmarks are based on the UMLS or its source vocabularies (like Snomed CT^35^), xMEN includes an implementation for easily obtaining such UMLS subsets. Given the configuration, the CLI command xmen dict creates the described UMLS subset, which can then be used through the Python API. It is also possible to pass a custom parsing script to create xMEN KBs from any terminology, including custom concept dictionaries or non-UMLS terminologies. For instance, we provide such scripts for the German OPS^37^ or the DisTEMIST gazetteer^45^ for our experiments. Indices into these KBs are precomputed using the xmen index command, which enables fast retrieval for CG. An example of a complete MEN pipeline for the Quaero corpus^23^ can be found in File S2. More detailed usage examples can be found in the source code repository.^46^

All operations in the xMEN toolkit are based on transformations of Hugging Face datasets built upon an extension of the BigBIO schema.^34^^,^^47^ Thus, any dataset that follows this schema and provides entity annotations is compatible with xMEN. In a real-world deployment, the input for entity normalization will usually be the output of a separate NER tagger. Converting offset-based span annotations produced by NER taggers to our input data format is straightforward. As an example, we provide an implementation for converting NER-tagged spaCy documents to BigBIO-compatible datasets as part of the xMEN package.^48^ The toolkit provides implementations for the described methods for NMT, abbreviation expansion, and other pre- and post-processing techniques, which can be used with any BigBIO-compatible dataset. Finally, the toolkit provides fine-grained evaluation metrics by integrating the widely used neleval tool.^49^ Given the flexibility of neleval, more relaxed metrics can be easily configured for different use cases (e.g., document-level scores).

### Benchmarks

To evaluate our approach, we use multiple BigBIO-compatible gold-standard datasets, as described in the following. More details regarding the annotation policy, target KBs, and the experimental protocol for each corpus can be found in File S1. All dataset-specific configurations are provided as Yaml files in the xMEN source code repository for reproducibility.^46^

#### Mantra GSC

The multilingual Mantra GSC comprises parallel text segments in 5 languages: English, Spanish, French, Dutch, and German.^26^ 100 bilingual Medline titles are available for English and each of the other languages, 100 sentences from European Medicines Agency (EMEA) drug labels in all 5 languages, as well as 50 parallel sentences from patents in English, French, and German. Entities have been annotated according to the Mantra terminology, a UMLS subset restricted to ten semantic groups and the source vocabularies MeSH,^50^  Snomed CT,^35^ and MedDRA.^51^ The Mantra GSC contains 5530 annotations, but much less in individual languages (e.g., only 1052 for the French subset). As baselines for our system’s performance on the Mantra GSC, we use the NMT-based system proposed by Roller et al,^29^ as well as CODER.^25^

#### Quaero

The Quaero corpus was used in the CLEF eHealth 2016 lab (task 2) and consists of 2 subsets: 2498 scientific articles from Medline and 10 drug monographs from the EMEA.^23^ Annotations in Quaero are based on the UMLS (2014AB version), restricted to 10 semantic groups. Annotation in Quaero has been carried out in a comprehensive fashion: nested entity spans are possible, and a single entity can be linked to multiple concept identifiers. The corpus provides 16 233 entity annotations, covering 5130 unique CUIs. Baselines are the NMT-based system proposed by Roller et al,^29^ as well as the distantly supervised and FS versions of MLNorm.^22^

#### BRONCO150

The German-language clinical corpus BRONCO^52^ consists of 200 de-identified oncological discharge summaries, 150 of which are publicly available through a data use agreement (BRONCO150). BRONCO was annotated with 3 entity classes and concept identifiers from corresponding German versions of the following terminologies: ICD-10 (*Diagnoses* annotations),^36^ OPS (*Procedures*),^37^ and ATC (*Medications*).^38^ In total, 4080 mentions of diagnoses, 3050 treatments, and 1630 medications have been annotated. Our baseline is the method proposed by Kittner et al,^52^ consisting of a dictionary lookup and rule-based re-ranking.

#### DisTEMIST

The DisTEMIST shared task was part of the 10th BioASQ lab.^45^ The dataset consists of Spanish-language clinical case reports from various medical specialties, annotated with disease mentions and a task-specific set of 111K Snomed CT concept codes, provided as part of the DisTEMIST gazetteer. In total, DisTEMIST has 8087 entity annotations linked to 3297 unique CUIs. As the best performance in the DisTEMIST competition was actually achieved by early prototypes of xMEN,^53^ we also compare with the second-best team (Better Innovations Lab^54^), as well as the mean and median across all teams in each task, according to the task overview paper.^45^

## Results

In the following, we share our experimental results. Tables 1-4 summarize the results for our four benchmark datasets and their subsets, through the different steps of CG, optional semantic type filtering (ST), and re-ranking. We refer to individual languages by their ISO 639-2/alpha-3 code, for example, *CE (WS*_Fre_*)* refers to the WS CE trained on the French translation of MedMentions. Whenever applicable, candidates are filtered based on semantic types before re-ranking to make results comparable to the baselines. Details on the quality of the NMT and label projection procedure can be found in File S3.

**Table 1.** F_1_@1 scores for the Mantra GSC through different steps of CG and after re-ranking with the WS CE for the respective language (CE (WS_{Language}_)).

	Medline					EMEA					Patents		
Language	Eng	Spa	Fre	Dut	Ger	Eng	Spa	Fre	Dut	Ger	Eng	Fre	Ger
**xMEN CG**													
TF-IDF	0.729	0.652	0.589	0.497	0.612	0.714	0.684	0.619	0.502	0.570	0.698	0.614	0.570
SapBERT	0.696	0.696	0.578	0.602	0.718	0.704	0.672	0.671	0.650	0.687	0.704	0.702	0.708
Ensemble	0.718	0.699	0.605	0.624	0.731	0.725	0.659	0.705	0.655	0.669	0.767	0.739	0.699
Ensemble + ST	0.833	0.769	0.705	0.683	**0.792**	0.806	0.754	0.759	0.728	0.741	0.816	0.771	0.760
**+ Re-ranking**													
CE (WS_{Language}_)	**↑0.869**	**↑0.838**	**↑0.756**	**↑0.713**	↓0.789	**↑0.827**	**↑0.789**	**↑0.766**	**↑0.730**	**↑0.753**	**↑0.857**	**↑0.834**	**↑0.799**

**Baseline**													
BTM^29^	–	0.691	0.674	0.614	0.663	–	–	–	–	–	–	–	–
GB^29^	–	0.687	0.686	0.648	0.679	–	–	–	–	–	–	–	–
CODER^25^	–	0.701	0.586	0.586	0.690	–	0.681	0.629	0.617	0.653	–	0.708	0.690

ST refers to semantic type filtering after CG. Precision and recall have been omitted due to space constraints, as they are almost identical to the F_1_ score for this dataset. Highest scores per column are highlighted **bold**, second-best underlined. Increased performance after re-ranking is indicated by **↑**, decreased performance by ↓.

**Table 2.** Benchmark results with FS and WS CE re-ranking for the Medline and EMEA subsets of the Quaero test set (CLEF eHealth 2016).

	Medline				EMEA			
	R@64	P@1	R@1	F_1_@1	R@64	P@1	R@1	F_1_@1
**xMEN CG**								
TF-IDF	0.787	0.563	0.562	0.563	0.720	0.554	0.552	0.553
SapBERT	0.920	0.621	0.620	0.621	0.876	0.571	0.570	0.571
Ensemble	**0.926**	0.583	0.582	0.583	0.879	0.573	0.572	0.573
Ensemble + ST	0.918	0.663	0.661	0.662	**0.892**	0.643	0.641	0.642
**+ Re-ranking**								
CE (WS_Fre_)	–	**↑**0.746	**↑** 0.746	**↑**0.746	–	**↑**0.711	**↑** 0.711	**↑**0.711
CE (FS)	–	**↑** 0.795	**↑0.780**	**↑** 0.788	–	**↑** 0.801	**↑0.781**	**↑0.791**

**Baseline**								
BTM^29^	–	0.771	0.663	0.713	–	0.781	0.692	0.734
MLNorm (DS)^22^	–	0.775	0.734	0.754	–	0.746	0.709	0.727
MLNorm (FS)^22^	–	**0.860**	0.740	**0.795**	–	**0.832**	0.670	0.743

ST refers to semantic type filtering after CG. DS and FS refer to the distantly supervised and fully supervised versions of MLNorm.  Highest scores per column are highlighted bold, second-best underlined. Increased performance after re-ranking is indicated by ↑, decreased performance by ↓.

**Table 3.** Benchmark results (5-fold cross-validation) for the different entity types in BRONCO150.

	Diagnosis				Treatment				Medication			
	R@64	P@1	R@1	F_1_@1	R@64	P@1	R@1	F_1_@1	R@64	P@1	R@1	F_1_@1
**xMEN CG**												
TF-IDF	0.790	0.548	0.520	0.533	0.379	0.080	0.072	0.076	**0.819**	0.526	0.494	0.510
SapBERT	0.890	0.639	0.638	0.639	0.544	0.210	0.192	0.201	0.784	0.481	0.452	0.466
Ensemble	**0.891**	0.648	0.647	0.648	**0.547**	0.209	0.191	0.199	0.805	0.437	0.410	0.423
**+ Re-ranking**												
CE (WS_Ger_)	–	↓0.628	↓0.628	↓0.628	–	↓0.185	↓0.180	↓0.183	–	**↑**0.580	**↑**0.569	**↑**0.574
CE (FS)	–	**↑0.807**	**↑0.746**	**↑0.775**	–	**↑0.748**	**↑0.411**	**↑0.530**	–	**↑0.807**	**↑0.684**	**↑0.740**

**Baseline**												
Kittner et al^52^	–	0.58	0.54	0.56	–	0.18	0.13	0.15	–	0.66	**0.68**	0.67

Highest scores per column are highlighted bold, second-best underlined. Increased performance after re-ranking is indicated by ↑, decreased performance by ↓.

**Table 4.** Benchmark results on the DisTEMIST shared task test set of the 10th BioASQ workshop.

	DisTEMIST			
	R@64	P@1	R@1	F_1_@1
**xMEN CG**				
TF-IDF	0.767	0.332	0.319	0.325
SapBERT	0.823	0.409	0.393	0.401
Ensemble	**0.830**	0.435	0.418	0.426
**+ Re-ranking**				
CE (WS_Spa_)	–	**↑**0.444	**↑**0.444	**↑**0.444
CE (FS)	–	**↑0.694**	**↑0.618**	**↑0.654**

**Shared Task**				
2nd Best Team^54^	–	0.548	0.458	0.499
Mean	–	0.402	0.331	0.353
Median	–	0.416	0.369	0.365

Highest scores per column are highlighted bold, second-best underlined. Increased performance after re-ranking is indicated by ↑, decreased performance by ↓.

### Candidate generation performance

The ensemble of TF-IDF and SapBERT achieves the best performance in recall@64 in most cases, which is the upper bound for recall of any subsequent re-ranking (up to 92.6% for Quaero). The only exception is the *Medications* subset of BRONCO, where the simple TF-IDF-based approach performs better by 1.4pp (Table 3). We attribute this to the fact that medication names are usually proper nouns with little morphological variability. At the same time, the list of aliases available for CG is very comprehensive through the inclusion of DrugBank, which is beneficial for TF-IDF.

The CG ensemble is also competitive regarding F${}_{1}@1$, making it a reasonable default choice if a re-ranker is unavailable. For Mantra GSC (Table 1) and Quaero (Table 2), where semantic type information is available, F${}_{1}@1$ is dramatically improved (up to 11.5pp) by restricting the candidate lists to concepts with compatible semantic types. In particular, we note that the type-filtered ensemble already improves upon all baselines for Mantra GSC, which can be further improved through re-ranking.

### Weakly supervised re-ranking

In most cases, our pretrained, WS re-ranking models improve performance over the ranking suggested by the CG ensemble. For the UMLS-based annotations in the Mantra GSC (Table 1) and Quaero (Table 2), the improvement is particularly strong. This is true even for the *EMEA* and *Patents* subsets of these corpora, although these are different text genres than the Medline abstracts in MedMentions, which have been used for pretraining. Weakly supervised re-ranking also improves performance over raw CG output for DisTEMIST and the *Medication* subset of BRONCO. This is surprising, as the target terminologies for these sub-tasks are different and do not even contain UMLS aliases in the case of BRONCO. However, for the *Diagnosis* and *Treatment* entities in BRONCO, the re-ranker trained on MedMentions slightly decreases performance. For these 2 scenarios, the target terminologies (ICD-10 and OPS) are comparatively small. Moreover, we use German aliases only, which is quite different from the aliases in the training dataset of the re-ranker. However, the negative impact of using an inappropriate pretrained re-ranker is relatively small, with up to 2pp decrease in top-1 performance for BRONCO (Diagnosis). For most entities in this scenario, the re-ranker just retains the ranking suggested by the CG.

### Fully supervised re-ranking

The FS re-ranker models perform best out of all xMEN configurations (when training data are available), achieving new state-of-the-art performance in nearly all cases, as we show in Tables 2-4. In particular, they outperform all baselines, except for the FS version of MLNorm,^22^ which obtains higher precision on both subsets of the Quaero corpus and a slightly higher $F_{1}$ score on the Medline subset (+0.7pp). The improvements through re-ranking over the strongest CG in terms of F_1_@1 are substantial for all datasets: the smallest improvement still amounts to +12.6pp for Quaero (Medline), and reaches up to +33.1pp for BRONCO (Treatments).

### Impact of rank regularization

**Figure 4.**
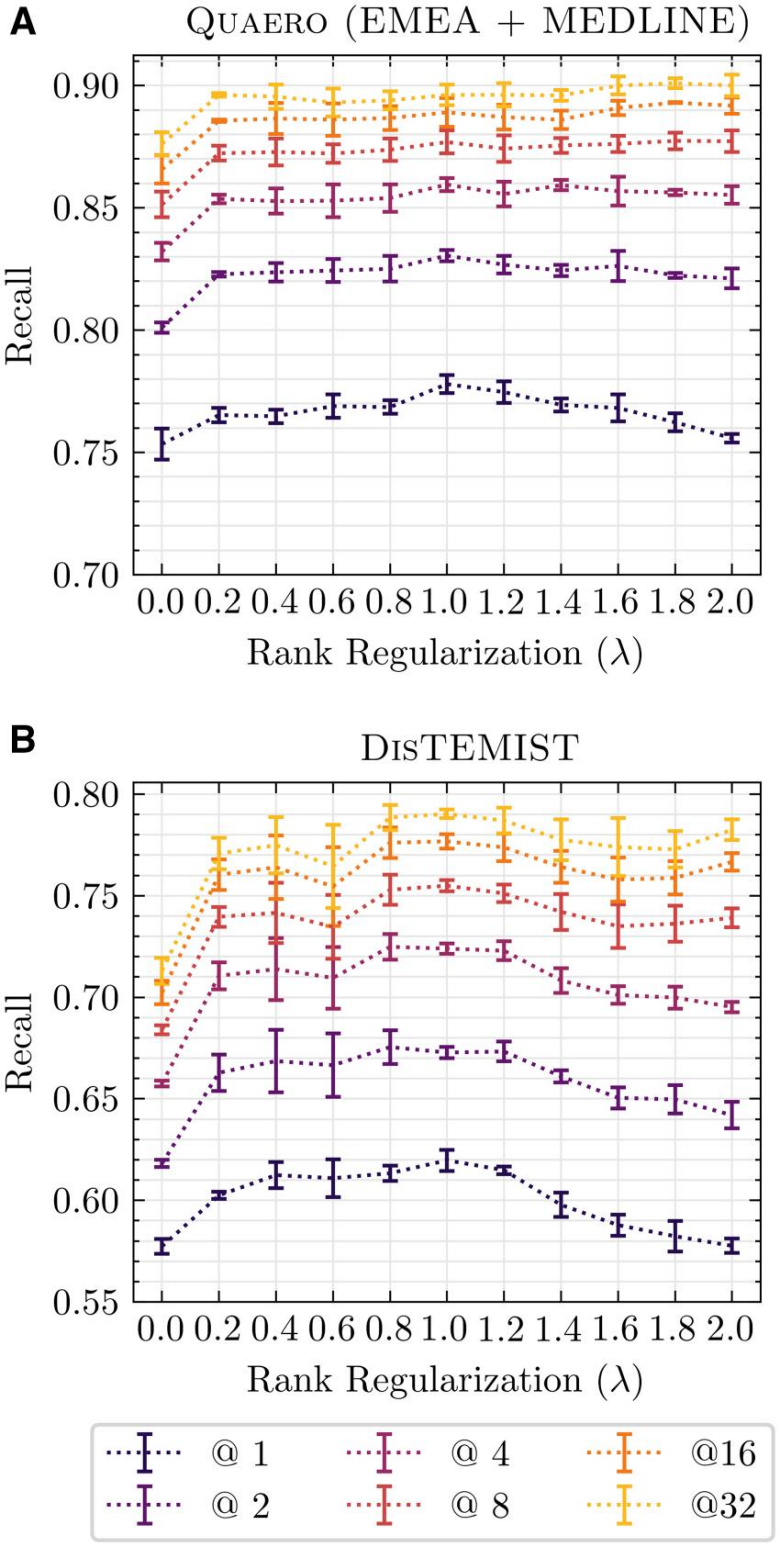
Impact of the relative weight λ of the rank regularization term in the CE loss function. We report the *test set* recall@*k* for different values of *k* in (A) for Quaero and (B) for DisTEMIST. For each value of λ, we report the mean and standard deviation across 3 runs with different random seeds. Note that the *y*-axes have different intervals, as the baseline performance is higher for Quaero.

During hyperparameter optimization, a value of λ=1 performed best on the validation sets of Quaero and DisTEMIST and was thus selected for all further experiments. Figure 4 shows an in-depth analysis on its performance impact. It is evident that recall@1 peaks for λ=1 also on the test sets for both Quaero and DisTEMIST, making it a sensible choice as a default value for CE training. However, nearby values in the range [0.4, 1.2] also seem to work well. For DisTEMIST, the differences between the optimal value and both no (λ=0) and too much (λ=2.0) regularization are more pronounced than for Quaero. Interestingly, the recall for larger values of *k* slightly improves for Quaero as regularization is increased. We assume that this is due to the suppression of NIL predictions when the initial CG ranking is given higher priority over the predicted ranking.

### Error analysis

Two conditions account for most false negatives, that is, reduced recall@1 during CG and subsequent ranking errors: complex entity mentions (consisting of multiple tokens) and lexical ambiguity of KB aliases. A more detailed quantitative analysis, as well as examples of the outputs produced by our pipeline and error conditions, are shown in File S4.

#### Complex entity mentions

For all analyzed corpora, recall@1 decreases for longer mention spans, both before and after re-ranking. Moreover, re-ranking can only successfully address these ranking errors for shorter entity spans. For instance, the FS CE trained on the Quaero training set improves recall from 0.659 to 0.816 (+15.7pp) for mentions of length one, from 0.639 to 0.710 (+7.1pp) for length 2, and from 0.625 to 0.674 (+4.9pp) for length 3. The results are similar for BRONCO, where recall values are lower overall, but re-ranking is mostly effective for mentions of length 1 or 2. However, the frequency of longer mention spans quickly decreases for both corpora, and most mentions are short. In comparison, DisTEMIST contains a larger fraction of long mention spans, with low recall values for spans of length greater than 2, which also hardly improve through re-ranking and hence have a large impact on the overall recall. Out of all mentions in DisTEMIST, where the CG step fails to retrieve the correct concept altogether, 75% consist of 3 or more tokens. This is in stark contrast to the other 2 datasets, where less than 14% (for BRONCO) and 10% (Quaero) of all CG errors are due to such complex mentions.

#### Lexical ambiguity

In our employed CG approach, concepts are retrieved based on independent aliases. Consequently, concepts sharing the same alias can be identified as a nearest neighbor match with the same candidate score. For instance, “lupus” is a valid alias for multiple UMLS concepts like “Lupus Erythematosus” or “Lupus Vulgaris,” so these concepts would have the same candidate score for a mention of “lupus” and the ambiguity needs to be resolved through re-ranking.

As the target KBs for BRONCO are very specific and mostly German, few aliases are shared among concepts (please consult File S4 for details). In most cases, the generated candidates share either zero aliases with the ground truth concept (i.e., when the concept was not retrieved as a candidate) or exactly one alias. For Quaero and DisTEMIST, where large, multilingual KBs are employed, lexical ambiguity is a major source of ranking errors. These errors can be effectively resolved through re-ranking. In contrast, re-ranking barely impacts performance@1 when the ground truth entity shares only a single alias with the generated candidates, that is, when the correct concept is retrieved as a candidate, but misranked for reasons other than lexical ambiguity.

#### Other errors

As the task of annotating grounded entity mentions is cognitively demanding, a certain degree of uncertainty due to annotation errors is also expected. For instance, the mention of “système nerveux” (*nervous system*) in Quaero was annotated with the semantic group *Anatomy*, but also with the concept “Malignant neoplasm of unspecified site of central nervous system” (C2977944), which does not belong to this semantic group.

## Discussion

In this section, we discuss the potential impact as well as the overall limitations of this work.

### Implications

Our proposed xMEN pipeline is designed to accommodate different levels of language-resource availability in real-world applications. The evaluation has shown that CG and pretrained re-rankers can transfer well across datasets and provide strong performance when a problem is reasonably similar to an existing, high-resource domain (e.g., UMLS-based annotations in MedMentions). However, our results also indicate that domain-specific models trained with gold-standard annotations are usually needed for state-of-the-art results. To obtain such data, xMEN can be easily integrated into linguistic annotation workflows, for example, by using candidate lists from pre-trained models as suggestions in annotation tools. For the popular INCEpTION annotation platform, we have already made such an integration available.^55^^,^^56^

Components of the xMEN toolkit also address critical gaps concerning the reproducibility in method development for MEN in many languages.^7^^,^^33^ By standardizing the definition of target KBs, data loaders, and evaluation metrics, it reduces variability and enables a consistent assessment across MEN systems, as these components of xMEN are independent of our specific, generate-and-rank approach.

### Limitations

While our approach was evaluated on multiple datasets covering diverse text genres, our selection is still limited to a rather small set of entity types and languages. A comprehensive collection of multilingual MEN benchmarks, covering more languages, is not yet available. The scarcity of gold-standard evaluation corpora for the MEN task, let alone large training datasets, appears to be even more pronounced for non-European languages.^6^^,^^8^ However, we hope that the flexible options for data loading, KB configuration, and evaluation protocols in xMEN will allow integrating additional benchmarks covering more diverse datasets and tasks from different domains, for example, more biologically oriented tasks, such as gene name normalization.^21^ The employed EasyProject method has been evaluated on 57 languages and can be easily applied to obtain additional WS re-ranking models. Future work could also consider alternative training/test splits in the benchmark datasets, which test the true zero-shot MEN abilities of systems realistically.^28^

In addition, xMEN could be further optimized in performance. For instance, at this point, we choose a single BERT checkpoint per language for initializing the CE models based on reported performance on other information extraction tasks.^57–59^ Although the chosen models perform well, it is possible that other pre-trained (e.g., multilingual or more domain-specific) encoders might perform better for the re-ranking problem.^60^ However, such ablation experiments were not the focus of the current work.

Our error analysis identified the primary sources of CG and ranking errors. While supervised re-ranking effectively resolves lexical ambiguity, we have yet to devise a strategy for enhancing overall recall and ranking for long entity mentions. This issue has predominantly affected the DisTEMIST corpus in our experiments, and has been less problematic for other corpora, presumably due to varying annotation policies (e.g., a preference for short vs long span annotations). Moreover, a certain number of less common normalization errors cannot be attributed to long mention spans or lexical ambiguity. A more detailed investigation of these errors might allow us to implement components for the xMEN toolkit to resolve them effectively.

## Conclusion

In the given work, we have presented a new approach and accompanying Python toolkit for normalizing medical entities in many languages. In particular, we combined general-purpose, unsupervised candidate generators with trainable CEs and a novel loss function for regularizing their training. We have extensively evaluated our system across a wide range of multilingual benchmark datasets.

The modularity of the xMEN system supports a broad practical applicability. In cases where no sufficient amount of annotated data for the target task and language are available for training, an ensemble of candidate generators with multilingual aliases already improves upon the state-of-the-art in numerous instances, for example, for the Mantra GSC or diagnoses and treatments in the BRONCO corpus. For UMLS-based or semantically related annotation schemes, a WS CE model pretrained on the MedMentions dataset can improve performance even further. State-of-the-art results are achieved for all benchmarks when task-specific training data can be used to train FS CE models with rank regularization.

There is ample opportunity to easily extend the xMEN toolkit with new modules, such as the integration of trainable candidate generators, using bi-encoders,^41^ clustering-based approaches,^15^ or generative models.^16^ Moreover, the SapBERT-based candidate generator can be quickly swapped with other models, which consider additional semantic relationships in their training procedures.^25^^,^^61^ Our error analysis has shown that long mention spans are particularly challenging to normalize, which might be alleviated by further pre-processing components that can be implemented within the xMEN framework.

In the future, we plan to evaluate our methodology on further English and non-English datasets once they become available to support MEN research. We believe that our configurable system for defining medical terminology subsets and implementation of evaluation metrics can form the basis for a comprehensive MEN benchmark, covering a diverse set of languages, text genres, and entity classes.

## Supplementary Material

ooae147_Supplementary_Data

## Data Availability

The benchmark results underlying this article are available on Dryad, at: https://doi.org/10.5061/dryad.15dv41p6h. The benchmark datasets are available on the Hugging Face Hub through the BigBIO project: https://huggingface.co/bigbio. The code for loading and pre-processing these datasets and re-produce our experiments is available as part of the xMEN toolkit. The version used in this work is available on Zenodo, at https://zenodo.org/records/12699190.
