# Supplementary material for: xMEN: a modular toolkit for cross-lingual medical entity normalization
Source: JAMIA Open. 2024 Dec 26;8(1):ooae147. doi: 10.1093/jamiaopen/ooae147 (PMC11671143; doi:10.1093/jamiaopen/ooae147)
Supplement: ooae147_Supplementary_Data [file ooae147_supplementary_data.zip › suppl1_Benchmarks_Experiments.pdf]

Supplementary File 1: Benchmarks and Experiments

Datasets

In the following, we describe the experimental setup for each benchmark dataset and derived target KBs in detail.

MANTRA GSC

Since the MANTRA GSC does not provide pre-defined train/validation/test splits, we do not train any fully supervised models for this dataset, but only evaluate the unsupervised CG and pre-trained CE models for each language. Target KBs for the MANTRA GSC are language-specific: the set of concepts consists of 681K UMLS CUIs with 1,904K English-language aliases. For all non-English languages, we add language-specific aliases for these CUIs, if available (between +32K aliases for Dutch and +1,054K aliases for Spanish).

QUAERO

Training, validation and test splits are provided for QUAERO, where the validation set of the 2016 dataset was the test set of the previous version (CLEF eHealth 2015 Task 1b). The target KB resulting from the task-specific UMLS subset consists of 2.91M CUIs, for which we use 6.91M aliases in French and English. For QUAERO, we initialize the BERT encoder of the fully supervised CE from the French biomedical BERT model DrBERT (4GB-CP-PubMedBERT) [1].

BRONCO

Within BRONCO150, five pre-defined cross-validation folds are provided, which we use to evaluate our models. For diagnoses and treatments, we follow the target KB definition from Kittner et al. [2] as closely as possible by using concepts and aliases from the German versions of ICD-10 and OPS, only. However, for medication names, the authors also report using the *Rote Liste* of drug names [3]. As this is a commercial database, we instead obtain trade names from the free resource DRUGBANK (version 5.1.10) to obtain additional (English) aliases for ATC codes [4]. The fully supervised CE model was initialized from the German biomedical model BIOGOTTBERT [5].

DISTEMIST

For DISTEMIST, we use the official training and test splits provided by the task organizers. As a validation set for model selection, we use a subset of 20% of the training set, which we have shown to be representative of the distribution of the held-out test set in earlier work [6].

For the 111K concepts in the official gazetteer, we obtain 1.52M Spanish and English aliases through the UMLS metathesaurus (2022AA). Here, we consider only UMLS concepts which can be mapped to one the SNOMED CT codes in the DISTEMIST gazetteer. The fully supervised CE is initialized from a Spanish biomedical-clinical RoBERTa model provided by Carrino et al. [7].

Experiments

For each benchmark dataset, we evaluate the performance of our different CG approaches. Therefore, we construct the target KB for each task and compute the indices for the TF-IDF- and SAPBERT-based CG. Then, we compute up to $k = 64$ candidates for each CG and the combined candidate lists. For a fair comparison with prior work, we use entity type information in QUAERO and the MANTRA GSC for CG [8, 9]. In particular, we use a semantic type filter after the ensemble CG step to restrict the candidate set to only those UMLS concepts consistent with the gold-standard semantic groups.

For all datasets, we take the top k candidates generated by the ensemble CG (plus optional semantic type filtering) and apply a weakly supervised cross-encoder *CE (WS)*, pre-trained on the entire MEDMENTIONS dataset, translated to the respective language. This step does not use any manually annotated training data from the target task. In addition, we train a fully supervised cross-encoder *CE (FS)* on the training splits of the respective datasets for all corpora, except MANTRA GSC. The CE models are trained for five epochs for MEDMENTIONS and 20 epochs for the other datasets, using a single NVIDIA A40 GPU and keeping the checkpoint that maximizes the F_1 score on each dataset's validation split.

Evaluation

Consistent with most prior work, we evaluate systems in terms of strict (span-level) precision, recall, and F_1 score, as well as recall@k for different numbers of retrieved candidates k. Our evaluation protocol needs to account for cases, where multiple candidates for a single entity mention are provided in the gold standard. This can happen for different reasons, e.g., when the mapping from mention span to concept is ambiguous and when multiple concepts occur inside the same mention span. For an in-depth discussion on the issue of multi-normalization, please refer to Ferré and Langlais [10]. In our experiments, we treat multiple gold concepts per mention as separate linkable entities, which requires fewer assumptions about the underlying annotation policy, but is stricter than other evaluation protocols [10, 11].

Model Selection and Hyperparameters

Our experiments use the default settings in the XMEN toolkit for all adjustable parameters. For instance, we use $k = 64$ candidates subject to re-ranking as suggested by Wu et al. [12], which also coincides with the batch size we can fit into 48 GB of GPU memory when training CE models. Beyond this batch size, recall only barely improves in our experiments.

In addition, we optimized the hyperparameters most relevant for training the CE through grid search using the validation sets of the DisTEMIST and Quaero corpora. These hyperparameters are the learning rate, context length, and our newly introduced rank regularization weight λ. We do not train any models specifically for the Mantra GSC, as it lacks designated training/test splits, so the corpus was not included in our grid search. Similarly, evaluation for BRONCO150 is based on 5-fold-cross-validation. Lacking a designated held-out test set, obtaining an unbiased performance estimate after hyperparameter optimization would only be possible through nested cross-validation, which was computationally not feasible for us.

The following hyperparameters resulted in the best validation set performance and have been used for all benchmarks:

- Learning rate = 2×10^{-5},
- Context length = 128 characters, and
- λ = 1.0 for rank regularization weight in the CE loss.

References

[1] Yanis Labrak, Adrien Bazoge, Richard Dufour, Mickael Rouvier, Emmanuel Morin, Béatrice Daille, and Pierre-Antoine Gourraud. DrBERT: A robust pre-trained model in French for biomedical and clinical domains. In *Proceedings of the 61st Annual Meeting of the Association for Computational Linguistics (Volume 1: Long Papers)*, pages 16207–16221, Toronto, Canada, July 2023. Association for Computational Linguistics.

[2] Madeleine Kittner, Mario Lamping, Damian T Rieke, Julian Götze, Bariya Bajwa, Ivan Jelas, Gina Rüter, Hanjo Hautow, Mario Sänger, Maryam Habibi, Marit Zettwitz, Till de Bortoli, Leonie Ostermann, Jurica Ševa, Johannes Starlinger, Oliver Kohlbacher, Nisar P Malek, Ulrich Keilholz, and Ulf Leser. Annotation and initial evaluation of a large annotated german oncological corpus. *JAMIA Open*, 4(2), April 2021.

[3] Rote Liste Service GmbH. Rote Liste, 2023. `https://www.rote-liste.de/` [retrieved: Oct 23, 2024].

[4] David S Wishart, Yannick D Feunang, An C Guo, Elvis J Lo, Ana Marcu, Jason R Grant, Tanvir Sajed, Daniel Johnson, Carin Li, Zinat Sayeeda, Nazanin Assempour, Ithayavani Iynkkaran, Yifeng Liu, Adam Maciejewski, Nicola Gale, Alex Wilson, Lucy Chin, Ryan Cummings, Diana Le, Allison Pon, Craig Knox, and Michael Wilson. DrugBank 5.0: a major update to the DrugBank database for 2018. *Nucleic Acids Res.*, 46(D1):D1074–D1082, November 2017.

[5] Manuel Lentzen, Sumit Madan, Vanessa Lage-Rupprecht, Lisa Kühnel, Juliane Fluck, Marc Jacobs, Mirja Mittermaier, Martin Witzenrath, Peter Brunecker, Martin Hofmann-Apitius, Joachim Weber, and Holger Fröhlich. Critical assessment of transformer-based AI models for German clinical notes. *Jamia Open*, 5(4):ooac087, November 2022.

[6] Florian Borchert and Matthieu-P. Schapranow. HPI-DHC @ BioASQ DisTEMIST: Spanish biomedical entity linking with pre-trained Transformers and cross-lingual candidate retrieval. In *Working Notes of Conference and Labs of the Evaluation Forum (CLEF). CEUR Workshop Proceedings*, pages 244–258, Bologna, Italy, 2022.

[7] Casimiro Pio Carrino, Joan Llop, Marc Pàmies, Asier Gutiérrez-Fandiño, Jordi Armengol-Estapé, Joaquín Silveira-Ocampo, Alfonso Valencia, Aitor Gonzalez-Agirre, and Marta Villegas. Pretrained biomedical language models

for clinical NLP in Spanish. In *Proceedings of the 21st Workshop on Biomedical Language Processing*, pages 193–199, Dublin, Ireland, May 2022. Association for Computational Linguistics.

[8] Roland Roller, Madeleine Kittner, Dirk Weissenborn, and Ulf Leser. Cross-lingual candidate search for biomedical concept normalization. *MultilingualBIO: Multilingual Biomedical Text Processing*, page 16, 2018.

[9] Perceval Wajsbürt, Arnaud Sarfati, and Xavier Tannier. Medical concept normalization in french using multilingual terminologies and contextual embeddings. *J. Biomed. Inform.*, 114:103684, 2021.

[10] Arnaud Ferré and Philippe Langlais. An analysis of entity normalization evaluation biases in specialized domains. *BMC Bioinformatics*, 24(1):227, 2023.

[11] Samuele Garda, Leon Weber-Genzel, Robert Martin, and Ulf Leser. BELB: a biomedical entity linking benchmark. *arXiv [cs.CL]*, 2308.11537, 2023.

[12] Ledell Wu, Fabio Petroni, Martin Josifoski, Sebastian Riedel, and Luke Zettlemoyer. Scalable zero-shot entity linking with dense entity retrieval. In *Proceedings of the 2020 Conference on Empirical Methods in Natural Language Processing (EMNLP)*, pages 6397–6407, Online, November 2020. Association for Computational Linguistics.
