# Supplementary material for: xMEN: a modular toolkit for cross-lingual medical entity normalization
Source: JAMIA Open. 2024 Dec 26;8(1):ooae147. doi: 10.1093/jamiaopen/ooae147 (PMC11671143; doi:10.1093/jamiaopen/ooae147)
Supplement: ooae147_Supplementary_Data [file ooae147_supplementary_data.zip › suppl4_Errors_Examples.pdf]

Supplementary File 4: Error Analysis and Examples

Quantitative Error Analysis

In Figure 1, we compare the (absolute) number of true positives for $k = 1$, i.e., the number of correctly predicted and ranked concepts for QUAERO, BRONCO, and DISTEMIST before and after fully supervised re-ranking. The "Total" line refers to the total number of concepts in the gold standard. The number of shared aliases in the right column is the maximum number of aliases that any concept in the candidate lists shares with the ground truth concept. When zero aliases are shared, this means that the correct concept was not among the retrieved candidates, therefore the number of true positives is also zero, before and after re-ranking.

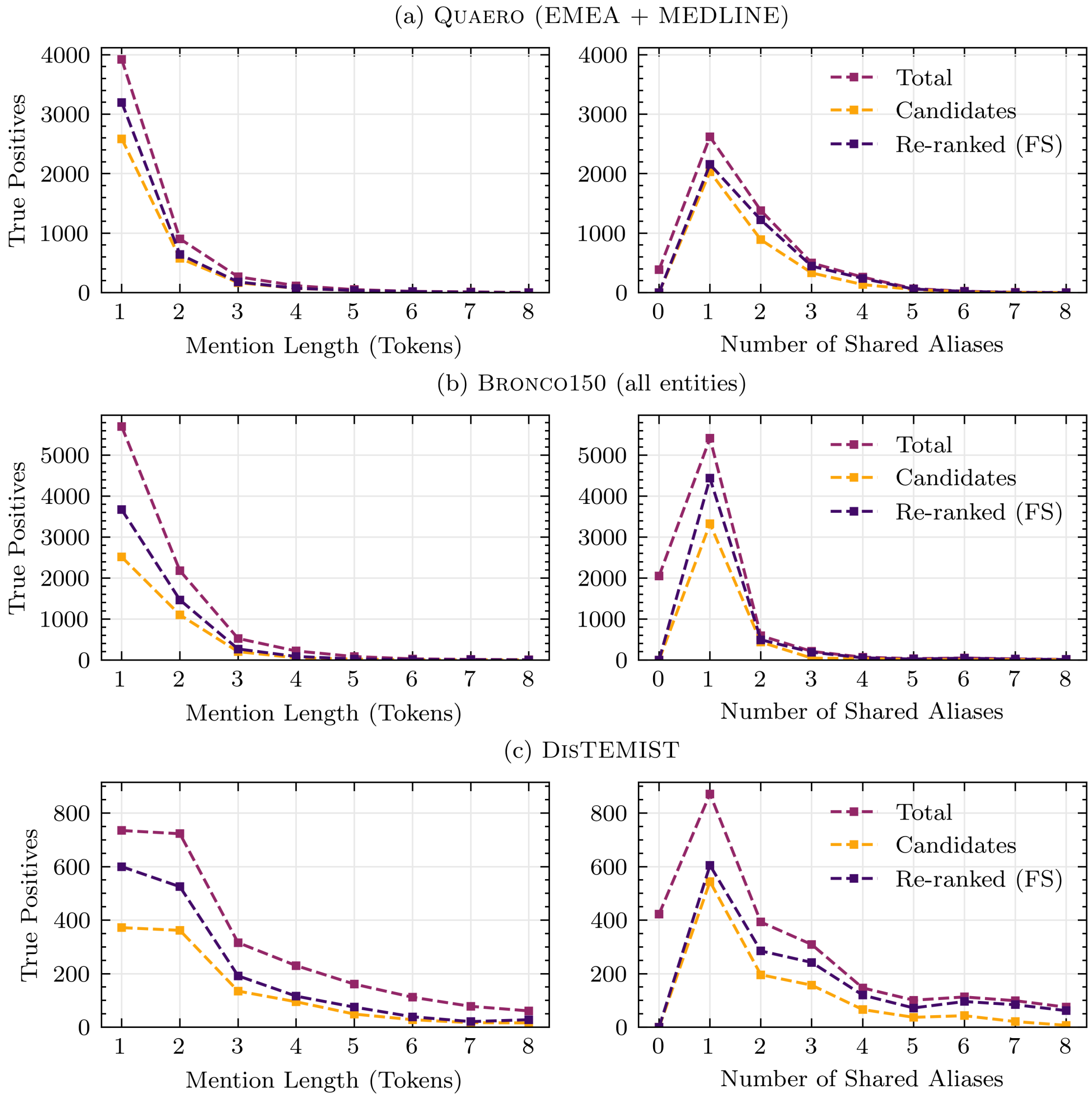

Figure 1: Quantiative Error Analysis

Qualitative Error Analysis

In the following, we provide examples for outputs produced by xMEN, demonstrating different error conditions and whether they can be recovered through re-ranking. All examples are taken from the French QUAERO corpus, with the respective entity mention highlighted in blue. The ground-truth concepts in the candidate lists before and after re-ranking are underlined.

Example 1

This example shows the normalization of an entity mention that is already correctly ranked by the candidate generator.

Input:
Deux nouvelles observations, soulignant la nécessité de l'étude ultrastructurale de la biopsie musculaire.

English Translation:
Two new observations, highlighting the need for ultrastructural study of muscle biopsy.

Candidates (Top 5) ✓	Re-ranked (Top 5) ✓
Biopsie (C0005558)	Biopsie (C0005558)
Biopsy Procedures on the Pharynx, ... (C3668914)	Consent Type - biopsy (C1548825)
Consent Type - biopsy (C1548825)	Biopsy Procedures on the Pharynx, ... (C3668914)
Surgical biopsy (C0565118)	Surgical biopsy (C0565118)
Biopsie par excision (C0184921)	Biopsie par excision (C0184921)

Example 2

This is an example of a more complex entity mention, which is also correctly ranked after candidate generation.

Input:
Hémangiomes choroïdiens circonscrits traités par photocoagulation au laser Argon.

English Translation:
Circumscribed choroidal hemangiomas treated with Argon laser photocoagulation.

Candidates (Top 5) ✓	Re-ranked (Top 5) ✓
Argon laser photocoagulation (C0185071)	Argon laser photocoagulation (C0185071)
Laser photocoagulation (C0441510)	Coagulation par laser (C0086524)
Argon laser coagulation Rx (C0947275)	Neodymium, yttrium, aluminum garnet laser ... (C0185074)
Consent Type - Laser Photocoagulation (C1548874)	Ion laser photocoagulation (C0185070)
Coagulation par laser (C0086524)	Krypton laser photocoagulation (C0185073)

Example 3

This is an example of a ranking error after candidate generation due to lexical ambiguity (shared aliases among candidate concepts), which can be resolved through re-ranking.

Input:
La lévofloxacine dans le traitement des pneumonies communautaires à pneumocoque.

English Translation:
Levofloxacin in the treatment of community-acquired pneumococcal pneumonia.

Candidates (Top 5) ✗	Re-ranked (Top 5) ✓
Pneumonite (C3714636)	Pneumopathie infectieuse (C0032285)
Pneumopathie infectieuse (C0032285)	Pneumonite (C3714636)
Pneumonie, organisme non précisé (C0339951)	Pneumonie, organisme non précisé (C0339951)
Pneumatose (C0333139)	Pneumopathie virale (C0032310)
Infective pneumonia (C0729704)	Pneumatose (C0333139)

Example 4

This example shows a ranking error after candidate generation for a complex entity with 6 tokens, which can be resolved through re-ranking.

Input:
Une solution buvable est également disponible pour le traitement des enfants de plus de 3 mois et chez les patients ne pouvant pas avaler des comprimés ou nécessitant une dose moins élevée.

English Translation:
An oral solution is also available for the treatment of children over 3 months of age, and for patients unable to swallow tablets or requiring a lower dose.

Candidates (Top 5) ✗	Re-ranked (Top 5) ✓
Enfants majeurs (C0683572)	Enfant d'âge préscolaire (C0008100)
Très grand prématuré (C3494262)	Nourrisson (C0021270)
Older child (C1455726)	Younger child (C0337547)
Enfant d'âge préscolaire (C0008100)	Enfant (C0008059)
Enfant (C0008059)	Young Child [Disease/Finding] (C0728836)

Example 5

This is an example of a ranking error, which cannot be fully resolved through re-ranking.

Input:
Approche endoscopique des lésions du troisième ventricule.

English Translation:
Endoscopic approach to lesions of the third ventricle.

Candidates (Top 5) ✗	Re-ranked (Top 5) ✗
Lésions du nerf oculomoteur (C1321926)	Lésions du nerf oculomoteur (C1321926)
Lésions du nerf trochléaire (C0161405)	Mass lesion of third ventricle (C2198307)
Lésions traumatiques du nerf optique (C0161398)	Mass lesion of fourth ventricle (C2198308)
Lésions du nerf trijumeau (C0161406)	Lésions encéphaliques (C0270611)
Mass lesion of third ventricle (C2198307)	Lésions du nerf vestibulocochléaire (C0161409)

Example 6

This case exemplifies a candidate generation error, where the correct candidate concept was not among the retrieved candidates and thus could not be re-ranked. The ground-truth concept is “Air Microbiology” (C0001867).

Input:
Contribution a l’ étude bactériologique de l’air a Paris.

English Translation:
Contribution to the bacteriological study of the air in Paris.

Candidates (Top 5) ✗	Re-ranked (Top 5) ✗
Test bactérien (C2242638)	Techniques bactériologiques (C0004642)
Techniques bactériologiques (C0004642)	Bacteriology–Technique (C3826675)
Bacteriology–Technique (C3826675)	Microbiota (procedure) (C3496041)
Analyse des bactéries dans le sang (C1167991)	Microbiology studies (C0947326)
Biotypage bactérien (C0085901)	Medical bacteriology–Technique (C3825231)
