# Supplementary material for: xMEN: a modular toolkit for cross-lingual medical entity normalization
Source: JAMIA Open. 2024 Dec 26;8(1):ooae147. doi: 10.1093/jamiaopen/ooae147 (PMC11671143; doi:10.1093/jamiaopen/ooae147)
Supplement: ooae147_Supplementary_Data [file ooae147_supplementary_data.zip › suppl3_Translation_Results.pdf]

Supplementary File 3: Translation Results

Table 1 shows the results of applying NMT and label projection to MEDMENTIONS. As expected, the entity alignment is imperfect, with up to 10.49% of labels that could not be recovered after the mapping (usually because of syntax errors, e.g., missing start or end tags). The loss for German and Dutch is much smaller—one reason might be that these belong to the same language family as the source language. We report the final test F_1 scores after CE training on the translated datasets as a sanity check. Except for English, these are substantially below the reported state-of-the-art results on the English MEDMENTIONS dataset (75.73% accuracy reported by Agarwal et al. [1]).

Table 1: Automatically translated versions of MEDMENTIONS, number of entities after label projection, and the relative loss in the number of entities compared to the source dataset. Furthermore, we report the test set F_1 score of the CE model trained for five epochs on these weakly labeled datasets.

Language	# Entities	Loss (%)	CE F_1
English (original)	203,282	-	.722
Dutch	200,231	1.50	.624
German	199,006	2.10	.598
Spanish	185,029	8.98	.569
French	181,958	10.49	.556

References

[1] Dhruv Agarwal, Rico Angell, Nicholas Monath, and Andrew McCallum. Entity linking via explicit Mention-Mention coreference modeling. In *Proceedings of the 2022 Conference of the North American Chapter of the Association for Computational Linguistics: Human Language Technologies*, pages 4644–4658, Seattle, United States, July 2022. Association for Computational Linguistics.
