# Supplementary material for: xMEN: a modular toolkit for cross-lingual medical entity normalization
Source: JAMIA Open. 2024 Dec 26;8(1):ooae147. doi: 10.1093/jamiaopen/ooae147 (PMC11671143; doi:10.1093/jamiaopen/ooae147)
Supplement: ooae147_Supplementary_Data [file ooae147_supplementary_data.zip › suppl2_xMEN_Toolkit.pdf]

Supplementary File 2: xMEN Components and Pipeline Example

Candidate Generation

TF-IDF with Character N-grams We encode all candidate aliases through character n-grams and compute TF-IDF vectors over the target KB to enable simple CG based on surface form similarity. For retrieval, we apply the same encoding to mention spans and rank candidates based on cosine similarity. Our implementation is adapted from SCISPACY, which we extended to be compatible with non-English UMLS subsets [1].

Dense Retrieval We implement a CG based on the cross-lingual version of SAPBERT to account for the semantic similarity between mentions and concepts [2]. Representations for aliases and mentions are based on the embedding of the `[CLS]` token in the last hidden layer of the BERT model. We use the cosine similarity to measure the similarity of concepts and mention embeddings. This component ensures high recall even when no aliases for a concept are available for the target language, but can be obtained from others, e.g., from the vast majority of English terms in the UMLS.

Ensemble To improve recall, we employ an ensemble of these well-established CGs, combining their scored candidate lists. These are merged and re-sorted according to their scores. As the scores are based on cosine similarity for both CG approaches, the resulting ranking is usually informative and does not require any re-weighting [3].

Candidate Ranking

Mention-context Encoding Each mention is encoded together with its context to the left and to the right (ctx_l and ctx_r). We extend the representation suggested by Wu et al. [4] to account for upstream abbreviation expansion and use the following representation:

`[CLS]` ctx_l `[START]` *mention* (*long form*) `[END]` ctx_r

with `[START]` and `[END]` denoting the beginning/end of the mention string. The context length is a configurable hyperparameter.

Concept Encoding We represent each concept by concatenating its canonical name with all its aliases ($Alias_{1..n}$). In addition to this representation (proposed by Xu et al. [5]), we also encode the concept's semantic type identifier to obtain the following representation:

semantic type `[TYPE]` *canonical name* `[TITLE]`
$Alias_1$ `[SEP]` ... `[SEP]` $Alias_n$

for example:

T047 `[TYPE]` Lupus Vulgaris `[TITLE]`
Lupus tuberculeux `[SEP]` Lupus exedens `[SEP]` Lupus vulgaire `[SEP]` ... `[SEP]` Tuberculosis cutis luposa

The encoding can be easily adapted to include other available concept metadata, like entity descriptions [6]. However, we did not consider these in our experiments, as these are not universally available in all KBs (and only for a subset of concepts in the UMLS).

Example Configuration for QUAERO

Here, we describe a minimal pipeline configuration for entity normalization using the QUAERO corpus. Code Listing 1 shows the configuration of the target KB, a subset of the 2014AB release of the UMLS metathesaurus, which was used for annotating the corpus. The KB is constructed using the CLI and the `xmen dict` command. Indices into this KB are pre-computed using the `xmen index` command. For candidate generation, the created indices are loaded and candidates are obtained from a BIGBIO dataset with entity spans, as shown in Code Listing 2. The candidates can be further processed, e.g., re-ranked with any of the pre-trained models. Instead of the pre-trained models, XMEN also allows training fully supervised re-ranking models.

Code Listing 1: Example configuration for the QUAERO target KB as a YAML file. The configuration can be used to reproduce the subset of concepts relevant for the QUAERO benchmark, where annotations are based on 10 semantic groups from the UMLS and concepts in the 2014AB release. To improve recall during candidate generation, we can obtain aliases for these concepts in different languages, here French and English.

```yaml
name: quaero
dict:
  umls:
    lang:
      - fr
      - en
    meta_path: ../2014AB/META
    semantic_groups:
      - ANAT
      - CHEM
      - DEVI
      - DISO
      - GEOG
      - LIVB
      - OBJC
      - PHEN
      - PHYS
      - PROC
```

Code Listing 2: PYTHON code for candidate generation, ranking, and evaluation using the example of the QUAERO dataset. The same pipeline can also be used for any dataset compatible with BIGBIO. Instead of the pre-trained cross-encoder, it also possible to train a supervised model based on the training split of the dataset.

```python
# Load dataset from Hugging Face Hub
import datasets
dataset = load_dataset("bigbio/quaero", "quaero_medline_bigbio_kb")

# Load knowledge base created with xmen dict
from xmen import load_kb
kb = load_kb("path/to/quaero.jsonl")

# Candidate generation
from xmen.linkers import default_ensemble
candidate_generator = default_ensemble(index_base_path="path/to/index")
candidates = candidate_generator.predict_batch(dataset, top_k=64)

# Post-processing
from xmen.data import SemanticGroupFilter
candidates = SemanticGroupFilter(kb).transform_batch(candidates)

# Load pre-trained cross-encoder
from xmen.reranking import CrossEncoderReranker
ce_dataset = CrossEncoderReranker.prepare_data(candidates, dataset, kb)
rr = CrossEncoderReranker.load("phlobo/xmen-fr-ce-medmentions", device=0)

# Prediction on test set
prediction = rr.rerank_batch(candidates["test"], ce_dataset["test"])

# Evaluation
from xmen.evaluation import evaluate
evaluate(dataset["test"], prediction)
```

References

[1] Mark Neumann, Daniel King, Iz Beltagy, and Waleed Ammar. ScispaCy: Fast and robust models for biomedical natural language processing. In *Proceedings of the 18th BioNLP Workshop and Shared Task*, pages 319–327, Florence, Italy, August 2019. Association for Computational Linguistics.

[2] Fangyu Liu, Ivan Vulić, Anna Korhonen, and Nigel Collier. Learning domain-specialised representations for cross-lingual biomedical entity linking. In *Proceedings of the 59th Annual Meeting of the Association for Computational Linguistics and the 11th International Joint Conference on Natural Language Processing (Volume 2: Short Papers)*, pages 565–574, 2021.

[3] Florian Borchert, Ignacio Llorca, and Matthieu-P Schapranow. Cross-Lingual candidate retrieval and re-ranking for biomedical entity linking. In Avi Arampatzis, Evangelos Kanoulas, Theodora Tsikrika, Stefanos Vrochidis, Anastasia Giachanou, Dan Li, Mohammad Aliannejadi, Michalis Vlachos, Guglielmo Faggioli, and Nicola Ferro, editors, *Experimental IR Meets Multilinguality, Multimodality, and Interaction*, pages 135–147, Cham, 2023. Springer Nature Switzerland.

[4] Ledell Wu, Fabio Petroni, Martin Josifoski, Sebastian Riedel, and Luke Zettlemoyer. Scalable zero-shot entity linking with dense entity retrieval. In *Proceedings of the 2020 Conference on Empirical Methods in Natural Language Processing (EMNLP)*, pages 6397–6407, Online, November 2020. Association for Computational Linguistics.

[5] Dongfang Xu, Zeyu Zhang, and Steven Bethard. A Generate-and-Rank framework with semantic type regularization for biomedical concept normalization. In *Proceedings of the 58th Annual Meeting of the Association for Computational Linguistics*, pages 8452–8464, Online, July 2020. Association for Computational Linguistics.

[6] Lajanugen Logeswaran, Ming-Wei Chang, Kenton Lee, Kristina Toutanova, Jacob Devlin, and Honglak Lee. Zero-Shot entity linking by reading entity descriptions. In *Proceedings of the 57th Annual Meeting of the Association for Computational Linguistics*, pages 3449–3460, Florence, Italy, July 2019. Association for Computational Linguistics.
